# Dementia Health Promotion for Chinese Americans

**DOI:** 10.7759/cureus.1411

**Published:** 2017-06-29

**Authors:** Benjamin K Woo

**Affiliations:** 1 UCLA

**Keywords:** chinese americans, dementia, health literacy, health promotion, knowledge dissemination, youtube, asian americans, health services, medical education

## Abstract

Introduction

This study aims to describe the results of a dementia awareness campaign in the Chinese American community.

Methods

The campaign consisted of a health fair, four dementia seminars, radio shows, television episodes, and a YouTube series. Descriptive statistics and qualitative data were obtained from various health communication channels.

Results

There were 156 and 313 participants in the health fair and dementia seminars, respectively. The participants in each component of the campaign also provided qualitative data on barriers and effective ways to disseminate awareness of dementia and brain health.

Conclusion

A dementia awareness campaign may be an effective way to reduce health disparities and dementia in the Chinese American community.

## Introduction

Dementia, also known as neurocognitive disorder, is a set of symptoms that include impairment of memory and executive functions [1]. While no current treatment can reverse the cognitive impairments, early diagnosis may allow patients and their families time to prepare for future changes and may prevent the use of costly medical resources [2]. Although executive dysfunction and memory loss can be delayed by educational programs on the benefits of memory training, physical activity, and a healthy diet, such programs are not well utilized in underserved communities [3]. Maximizing community-academic partnerships through cooperative efforts among academia, community agencies, and individuals may be necessary to increase health promotion programs [4-6]. However, little is known about how to best educate minority groups on dementia and dementia prevention.

As a minority group, Chinese Americans are at especially high risk for delayed diagnosis and suboptimal management of dementia [7-10]. Chinese patients often delay seeking dementia care until the severe stages of neuropsychiatric symptoms when compared to Caucasian patients [7]. Among elderly Asian Americans, under-detection of cognitive impairment remains prevalent [8]. Following a diagonisis of neurocognitive disorder, Asian American patients were less likely to receive dementia treatment than their white counterparts [9]. Moreover, while clinical depressive symptoms were more common among Chinese American dementia patients, they were less likely to receive antidepressant therapy than white subjects [10].

The intense stigma associated with mental illness in Chinese culture, a lack of dementia-related knowledge, and poor availability of culturally sensitive information about dementia are some important factors that may dissuade Chinese Americans from seeking help for dementia [5,11]. Approximately one out of every four elderly Chinese Americans stigmatized dementia as a mental illness [12-13]. Chinese Americans also have more negative perceptions toward dementia than other chronic illnesses [14]. Many Chinese Americans, especially those who are acculturated, hold a misconception that dementia does not impact life expectancy [15]. Even the family members of patients with dementia tend to have moderately stigmatizing views about dementia in the Chinese American community [16]. Despite this high level of stigma, only 13% of Chinese American immigrants were aware of dementia services in their community [11]. Studies have also shown that Asian Americans may have low levels of biomedical understanding of dementia due a to lack of culturally appropriate materials [6,17-18]. Nevertheless, despite holding misconceptions and being less knowledgeable about dementia, Chinese Americans are eager to participate in dementia educational campaigns [19-20].

While research has examined how to craft and deliver health messages in general, very little research focused on how to deliver broader public health campaigns to the general public of Chinese Americans. Studies have furthermore shown that low dementia health literacy remains common in the Chinese American community, even among the younger generation of family members of patients with dementia [21-22]. As such, promoting dementia understanding and culturally sensitive dialogues is necessary to improve health literacy for the Chinese American community. In this article, we provide an overview of how collaborative efforts are necessary to achieve dementia awareness among Chinese Americans. We also examine each communication media format separately to highlight how seemingly trivial concerns could significantly affect health knowledge dissemination.

## Materials and methods

The project began in 2012 as a partnered, participatory planning program that was headed by a leadership group that included representatives from each of the four stakeholder perspectives: academia, the community, community service providers, and faith-based organizations, with the goal of improving the well-being of Chinese American older adults. These partners included the Asian Pacific Health Corps (APHC) at the University of California, Los Angeles (UCLA); Chinatown Service Center; Alzheimer’s Association, California Southland Chapter; Chinese Outreach; Herald Crusades; KMRB AM1430 Radio Station; Phoenix Satellite Television Cantonese News; Lutheran Church of the Holy Spirit; Chinese Bible Missions Church; and Los Angeles Chinese Alliance Church. Over time, additional agencies, including Alzheimer’s Association Northern California Chapter and Faith Lutheran Church, became involved. The goals were to promote dementia awareness and to begin a conversation about dementia among the Chinese American community so that the community may better recognize cognitive impairments.

The work group held outreach events at different venues to improve the Chinese American general public’s ability to recognize neurocognitive disorders. One of the first outreach events sponsored by the APHC at UCLA was a health fair hosted in the Los Angeles Chinatown. Despite several initial setbacks, other dementia community outreach events were set up, which led to message delivery through multiple media channels. Additional community outreach efforts included Cantonese seminars, radio shows, television episodes, and a YouTube series. The ultimate goal was to increase Chinese Americans’ knowledge about the causes and risks of dementia. We used descriptive statistics to summarize the demographic information of the participants. We also provided qualitative data in the form of various comments from the general public.

## Results

Health fair

In May 2012, 156 Chinese-speaking participants attended the UCLA APHC Chinatown health fair. A total of 95 elderly (age ≥ 65 years old) subjects were included in the statistical analysis. The average age of the participants was 77.5 years (SD = 7.0), and 65.3% (*n *= 62) of them were women. 41.1% (*n *= 39) were high school graduates, and 91.6% (*n *= 87) were retired. 11.6% (*n *= 11) spoke only Chinese.  In the past three months, 93.7%  participants (*n *= 89) had not visited their primary care physicians.

While dementia education was provided during the health fair, elderly participants were instead more interested in the following services: physician consultation, osteopathic manipulative treatment, and bone density screening. In the health fair, the dementia education service quickly became medical encounters. Elderly participants were only interested in resolving their immediate medical concerns with health professionals. Chinese-speaking participants were more interested in medical than psychiatric care. While it helped to have patient educational material on dementia, one participant stated in Chinese, “Just teach us how to prevent memory loss!” Another participant said, “I don’t read, how can I understand these papers?”​​

Seminars

There were 313 Chinese-speaking participants in a total of four free-of-charge seminars on dementia awareness, held in California. A total of 66.1% (*n *= 207) were females, 53.0% (*n *= 166) had completed high school, and 51.1% (*n *= 160) had immigrated to the United States less than 20 years ago. While only 30% (*n* = 94) of the participants were elderly, over 72.5% (*n *= 227) were older than 40 years old. As these individuals would be entering the age group at risk for dementia, improving dementia literacy would be timely for such participants.

Each one of the four presentations was one hour long, followed by 30 minutes of questions and answers (Q&A). In one of the dementia seminars, 50 participants submitted an optional survey to indicate the means by which they became aware of the seminar. The most frequent means were church announcements and posters (34.0%) and word-of-mouth from the community (26.0%) [19].  

During one of the Q&A sessions, three participants asked the following questions. One participant inquired, “What questions should I ask to get my father tested for memory problems?” A member also asked, “What other vitamins or natural supplements do you recommend to prevent memory loss?” Another individual questioned, “What else can we eat to slow dementia?”​

Radio shows

The Cantonese radio program, *Rainbow Beneath the Sky*, is broadcasted on KMRB AM1430 in the Greater Los Angeles area [4]. On June 12, 2012, a one-hour dementia awareness campaign was aired. The details of the radio show and the findings have been described elsewhere [23].

Engagement strategies, such as use of celebrity stories and their battles with dementia, appeared to engage Chinese Americans to call in to the radio show. As one participant shared, “Playing mah-jongg can eliminate dementia because it activates my brain!” [23]. Furthermore, the radio host shared her own personal story on caring for her mother with dementia.

The radio show host and audience members (via e-mails) made the following recommendations for promoting dementia awareness in the Chinese American community: (1) use patient stories as a starting point; (2) include a checklist of questions for the general public to ask their doctors; (3) discuss all relevant treatments and spell out all medication names; and (4) provide up-to-date statistics about dementia.​

Television episodes and YouTube series

The North American Chinese Phoenix Satellite Television station aired two 30-minute dementia educational episodes in January 2014. Part one covered general dementia knowledge, including symptoms, staging, risk factors, etiology and different types of dementia such as Alzheimer’s disease. The second part focused on dementia diagnosis and prevention, educating viewers on the different elements of the mental status examination, potential unrecognized medical illnesses that can cause or exacerbate dementia, and the importance of healthy lifestyles. Subsequently, both real-time recordings were uploaded to YouTube as two 25-minute videos in the Cantonese language.

After a 12-month period, viewing data for the second episode (dementia diagnosis and prevention) were collected from YouTube for analysis. The YouTube video generated a total of 625 views, resulting in 4,354 minutes of watch time. In terms of watched time (minutes), the top five geographical locations were: Hong Kong (2287), United States (702), Singapore (296), Australia (281), and Canada (220). The average view duration was seven out of 25 minutes. In terms of viewing devices, 40%, 32%, and 23% viewers watched the episode from computers, mobile phones, and tablets, respectively.

The television host and YouTube viewers (via e-mails) made the following suggestions for increasing dementia knowledge in the Chinese American community: (1) walk the audience through a family member noticing loved one’s memory loss; (2) discuss how to convince loved ones with cognitive impairments to see a doctor; (3) remind the audience of the availability of Chinese translator services available to patients and families; and (4) emphasize that Chinese Americans are at risk for dementia.

## Discussion

As increasing knowledge of dementia can lead to changes in health-related behaviors, dementia awareness campaigns are important and necessary. This article helps to better understand why collaborative efforts are necessary to promote brain health in the Chinese American community. The results show that various media channels can be used to disseminate health education amongst Chinese Americans. However, there are also challenges in educating this population about neurocognitive disorders. We identified several barriers and effective ways to address them in dementia knowledge dissemination among Chinese Americans.

As health fairs expose Chinese immigrants to the need to obtain health services, dementia education materials can be distributed as a first step to reduce health care disparity. However, one of the barriers endorsed by health fair participants was the lack of culturally competent resources and patient education materials in written Chinese. This finding indicates that in order to effectively disseminate knowledge in a health fair, more needs to be done to ensure the readability of translated patient education materials. Over 60.0% of our health fair participants were senior immigrants, and 58.9% did not graduate from high school. Previous studies have found evidence that Chinese immigrants with low levels of health literacy suffered from low levels of health status [24-25]. Our study adds that improved health literacy may require verbal instruction to supplement written patient education materials, especially for Chinese American immigrants who speak little or no English.

Health promotion is necessary to train Chinese immigrants to lead healthy lifestyles to prevent dementia. Approximately three out of four dementia seminar participants were entering the age group of possible dementia development. This finding indicates that health seminars as communication channels were effective in targeting the population at risk. Furthermore, as this intervention targeted members of the lay community, we were able to demonstrate the importance of having a community-based event consisting of both lecture and Q&A components to promote public awareness about neurocognitive disorders in the Chinese American community. This study identifies the interest that the participants in these seminars had in further learning the facts, myths, and controversies surrounding dementia. This is consistent with descriptions in previous literature: when presented with culturally appropriate educational opportunities, Chinese Americans were interested in learning more about dementia [19].

Mass media plays an important role in disseminating dementia information to the Chinese American general public. While we were unable to quantify the impacts of utilizing an ethnic radio station, previous research has indicated that about 50% Chinese American immigrants used radio to access health information [26]. This study demonstrates that in addition to obtaining knowledge, community members were willing to call in to a radio show in response to a dementia awareness campaign. As such, it shows that radio shows have the potential to effectively promote health knowledge in the Chinese community. The audience identified information overload as a communication barrier, especially for a one-hour radio show. One way to reduce this barrier is to provide a checklist of important information for radio show listeners. Last but not least, the use of personal stories from dementia patients or caregivers could further promote brain health among this target group.

As the key priorities of the campaign were to create public awareness and to raise support in the Chinese American general public for dementia awareness, both television episodes and the YouTube series were effective in reaching a larger audience. In fact, YouTube extended the audience beyond the local community into other Chinese-speaking regions as well [27]. Viewers believed that emphasizing how Chinese Americans are at risk for dementia would make it easier to discuss dementia. In addition to educating Chinese Americans on the signs and symptoms of the disease, other health campaigns may want to highlight the prevalence of the disease to this vulnerable population. Our results suggest that providing solutions to defeat dementia may reduce barriers to knowledge dissemination in the Chinese community. Future dementia awareness campaigns should further focus on practical ways to care for and communicate with possible cognitively impaired patients. Such steps would be necessary to reduce health disparities and dementia among Chinese Americans.

Previous research has demonstrated that Chinese Americans elders can be successfully recruited into dementia research [5]. Our dementia awareness campaign, which utilized multiple culturally sensitive health communication channels, proved successful in promoting brain health among Chinese Americans. The present study has some limitations. First, data were cross-sectional and were based on descriptive characteristics without any pre-post comparisons. Therefore, whether participants actually retained dementia knowledge from the campaign cannot be answered by this study. Second, there were no measures of acculturation. Third, it was impossible to know from this study whether participants have other channels or ways to further educate themselves on dementia. Nevertheless, the present study has identified barriers as well as creative ways to disseminate dementia knowledge among Chinese Americans. Educational interventions that are delivered in native Chinese languages and in a culturally sensitive manner are needed to effectively raise dementia awareness in Chinese community. These findings call for collaborative efforts among different community stakeholders in order to defeat dementia. Future health awareness campaigns should further focus on mass media, especially with the use of the Internet, to reach a broader audience of Chinese Americans.

## Conclusions

This study described the results of a dementia awareness campaign in the Chinese American community. The campaign consisted of a health fair, four dementia seminars, radio shows, television episodes, and a YouTube series. The results show that various media channels were important and necessary to disseminate health education amongst Chinese Americans. Furthermore, collaborative efforts would be necessary to reduce dementia and health disparities in the Chinese American general public.
